# Whole-Exome Sequencing Revealed Mutations of *MED12* and *EFNB1* in Fetal Agenesis of the Corpus Callosum

**DOI:** 10.3389/fgene.2019.01201

**Published:** 2019-11-25

**Authors:** Ying Jiang, Ye-Qing Qian, Meng-Meng Yang, Qi-Tao Zhan, Yuan Chen, Fang-Fang Xi, Matthew Sagnelli, Min-Yue Dong, Bai-Hui Zhao, Qiong Luo

**Affiliations:** ^1^Department of Obstetrics, Women’s Hospital, School of Medicine, Zhejiang University, Hangzhou, China; ^2^University of Connecticut School of Medicine, Farmington, CT, United States

**Keywords:** whole-exome sequencing, *de novo* mutation, fetal agenesis of the corpus callosum, Sanger sequencing, prenatal diagnosis

## Abstract

Agenesis of the corpus callosum (ACC) is a birth defect in which the corpus callosum is either partially or completely missing. With recent advances in prenatal ultrasound, detection of ACC in obstetric practices is becoming more common. Etiologies of ACC include chromosome errors, genetic factors, prenatal infections, and other factors related to the prenatal environment. In an effort to elucidate more about the genetic influence in the pathogenesis of ACC, we identified, through whole-exome sequencing (WES), two gene mutations in two families with complete agenesis of the corpus callosum. These two mutations are located on chromosome X: one is a hemizygous missense mutation c.3746T>C (p. L1249P) in the gene mediator complex subunit 12 (*MED12*); the other one is a heterozygous missense mutation c.128+5G>C in gene ephrin B1 (*EFNB1*). Historically, early diagnosis of complete ACC during pregnancy has been difficult; however, WES has provided us with a creative avenue of diagnosis, combining identification of genetic mutations with prenatal imaging.

## Introduction

The corpus callosum (CC) is one of the five main cerebral commissures connecting the left and right cerebral hemispheres. Corpus callosum abnormalities are separated into three categories: complete agenesis, partial agenesis, and dysgenesis (Palmer and Mowat, 2014). The prevalence of agenesis of the corpus callosum (ACC) is around 1.4 per 10,000 live births (Glass et al., 2008). ACC may be associated with intellectual disabilities, epilepsy, behavioral difficulties, and cognitive impairments (Moutard et al., 2003). Many couples decide to terminate the pregnancy on the basis of ultrasound (US)/magnetic resonance imaging (MRI) findings in ACC. However, follow-up studies indicate that 71.2% of isolated ACC cases have normal intelligence, 13.6% have borderline or moderate intellectual disabilities, and 15.2% have severe intellectual disability (Santo et al., 2012). These findings highlight the importance of improving the precision of the diagnosis of ACC as well as moving the window of diagnosis earlier in pregnancy.

Studies have shown that the majority of ACC cases are caused by *de novo* dominant mutations (De Ligt et al., 2012). The Netrin receptor *DCC* (*Deleted in colorectal cancer*) gene is a critical factor in corpus callosum development in mice and works through orienting callosal axons at the midline (Fazeli et al., 1997). *DCC* mutations have been shown to cause isolated ACC, including the reported mirror movement (MM) phenotype, in humans (Marsh et al., 2017). Additionally, studies have shown that fibroblast growth factor receptor/glial fibrillary acidic protein (*Fgfr1/Gfap*) play an important role in corpus callosum formation (Smith et al., 2006). Recently, whole-exon sequencing has provided the opportunity for molecular genetic screening of rare human diseases.

In the present study, we performed whole-exome sequencing in two families who had children who were affected by complete ACC. With whole-exon sequencing and Sanger sequencing, we identified two single-nucleotide missense mutations which are likely related to the pathogenesis of ACC.

## Clinical Report

### Proband 1

**Figure 1:**
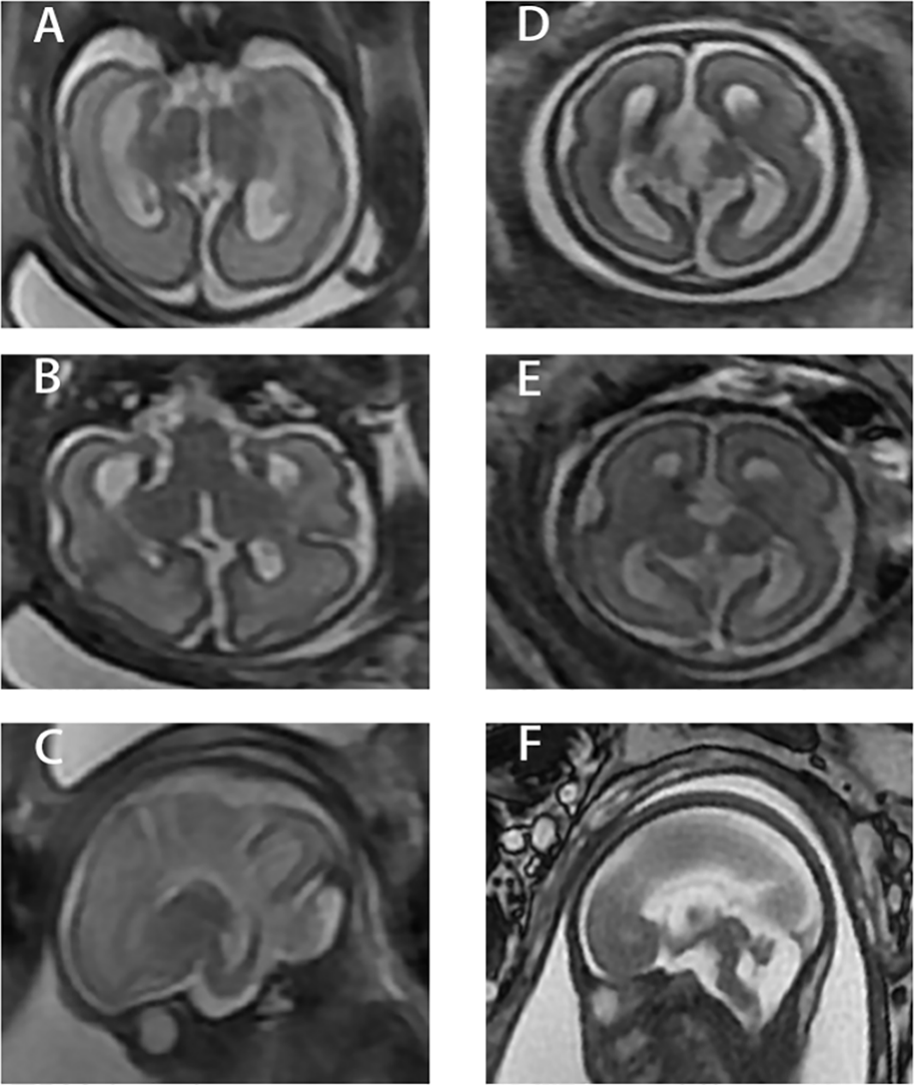
Three different sections of the two cases. **(A**–**C)** Case 1. **(D**–**F)** Case 2. 1) **(A)** and **(D)** are coronal images from fetal MRI. 2) **(B)** and **(E)** are axial images from fetal MRI. 3) **(C)** and **(F)** are sagittal images from fetal MRI.

A fetal ultrasound of a 21-year-old G1P0 female at 26 weeks gestation revealed agenesis of the corpus collosum and a right lateral ventricle about 1.0 cm in width. A subsequent brain MRI performed at 28 weeks and 5 days gestation, determined by last menstrual period (LMP), confirmed complete callosal agenesis and showed mild ventriculomegaly. Both lateral ventricles had a width of approximately 11 mm, were parallel, and teardrop-shaped. The third ventricle was slightly widened and uplifted. No obvious corpus callosum signals were observed in each section. The cisterna magna was about 9 mm in width, and the vermis cerebelli was present (**Figures 1A–C**). A female fetus was delivered at 31 weeks. There is no anatomic pathology data for the fetus.

### Proband 2

A 33-year-old G1P0 female presented at 26 weeks gestation for a fetal ultrasound which demonstrated agenesis of the corpus callosum. A subsequent brain MRI performed at 27 weeks, determined by LMP, confirmed complete callosal agenesis complicated by variant Dandy–Walker malformation. Specifically, the posterior horn was about 0.6 cm in width, the bilateral lateral ventricles were parallel, and the third ventricle was widened and uplifted. There was no obvious corpus callosum in each section. The posterior cranial fossa was about 0.8 cm in width and the fourth ventricle was about 0.9 cm in width. The superior vermis was present while the lower vermis was absent (**Figures 1D–F**). The male fetus was delivered at 27 weeks, 5 days gestation. There is no anatomic pathology data for the fetus.

Chromosomal array (CMA) result of both the probands using CytoScan™ HD whole-genome SNP array (Affymetrix, USA) showed no disease-related copy number variations (CNVs). The reporting threshold of the copy number result was set at 500 kb with marker count ≥50 for gains and 200 kb with marker count ≥50 for losses.

## Materials and Methods

### Ethics Approval for the Investigation

The Ethics Committee of the Women’s Hospital, Zhejiang University, School of Medicine (Hangzhou, China) approved our study. The investigation conforms to the principles outlined in the Helsinki Declaration. Written informed consent was obtained from the patients and their spouses whose child’s tissue was collected.

### DNA Extraction

A 5-ml aliquot of peripheral whole blood was collected from the parents and EDTA was added for anticoagulation. Additionally, a 1 × 1 × 1-cm medial thigh muscle sample was gathered from the induced fetus. Genomic DNA from 2 ml of peripheral whole blood was extracted using a Qiagen Blood DNA mini kit (Qiagen^®^, Hilden, Germany). DNA from muscle was extracted with a Genomic DNA Purification Kit (Invitrogen, cat. K0512, USA) and then preserved at -20°C. The remaining samples were stored at -80°C.

### Whole-Exome Sequencing and Data Analysis

Target enrichment of target region sequences was carried out by the Agilent SureSelect Human Exon Sequence Capture Kit. The sequencing libraries were quantified using the Illumina DNA Standards and Primer Premix Kit (Kapa Biosystems, Boston, MA, USA), massively parallel sequenced using the Illumina HiSeq 2500 platform (Illumina, San Diego, CA, USA), and then massively parallel sequenced again using the Illumina HiSeq 2500 platform (Illumina, San Diego, CA, USA). After sequencing and filtering out low-quality reads, high-quality reads were compared to the human genome reference sequence (GRCh37.p12, hg19). The GATK software was used to call variants.

### Data Filtering

In our study, we selected variants in fetuses by complying with the following criteria: 1) mutation frequency ≥0.01, quality <300, and alt/depth <25%; 2) MAF > 0.02 when referring to dbSNP (https://www.ncbi.nlm.nih.gov/projects/SNP/), the 1000 Genomes Project (http://www.1000genomes.org/), and the ExAC Browser (http://exac.broadinstitue.org/); 3) variants located in deep intron regions; 4) variants reported benign by ClinVar (https://www.ncbi.nlm.nih.gov/clinvar/); and 5) depth >20 and alt/all <0.1. Subsequently, the variants left were further selected according to the following criteria: 1) deleterious protein or splicing predictions; 2) variants in a gene responsible for an Online Mendelian Inheritance in Man (OMIM) disease/phenotype associated with the probands; and 3) disease inheritance models: genes associated with autosomal-recessive disease (AR), defined as variants in homozygous or compound heterozygous fetuses with parents who are both heterozygous carriers; genes associated with autosomal-dominant disease (AD), defined as *de novo* variants in fetuses or variants inherited from either of the parents for non-lethal or late-onset diseases; genes associated with X-linked recessive disease (XLR), characterized by hemizygous male fetuses with heterozygous mothers; and genes associated with X-linked dominant disease, characterized by *de novo* variants in fetuses or variants in fetuses that were inherited from either of the parents in the case of non-lethal or late-onset disease. The identified novel mutations were further assessed for possible pathogenicity using PolyPhen-2, SIFT, PROVEAN, HSF, regSNP-intron, and MutationTaster. The selected variants were classified by following the American College of Medical Genetics and Genomics/Association for Molecular Pathology (ACMG/AMP) guidelines. All putative disease-causing variants detected by new-generation sequencing (NGS) were confirmed in probands and their parents by Sanger sequencing. Sequencing was performed on ABI 3500xL (Applied Biosystems, Foster City, CA, USA), and the results were analyzed using DNASTAR software.

## Results

### Identification of Variants on Two Probands

In proband 1, with the average coverage of 132.28X on targeted regions, we confirmed 95,687 novel genetic variants (GVs) through whole-exome sequencing (WES), while for proband 2, under mean coverage of 71.63X, we identified 79,974 GVs. The summary of WES for the two probands is shown in **Table 1**.

**Table 1:** Summary of whole-exome sequencing on these two probands.

	Proband 1	Proband 2
**Total captured region size**	60 M	60 M
**On-Target Reads (%)**	73.98	72.6
**% of captured regions with coverage >20**	98.6	96.26
**Mean coverage of target region**	132.28	71.63
**Total number of SNPs**	348,832	218,408
**Total number of INDELs**	37,565	21,246
**Total number of novel GVs not listed in dbSNP**	95,687	79,974
**Total number of SNPs in a panel of corpus callosum agenesis-related genes**	23	11
**Total number of INDELs in a panel of corpus callosum agenesis-related genes**	1	3

SNP, single-nucleotide polymorphism; INDEL, insertions and deletions; GV, genetic variants.

### Pathogenic Mutations on Two Probands

**Figure 2:**
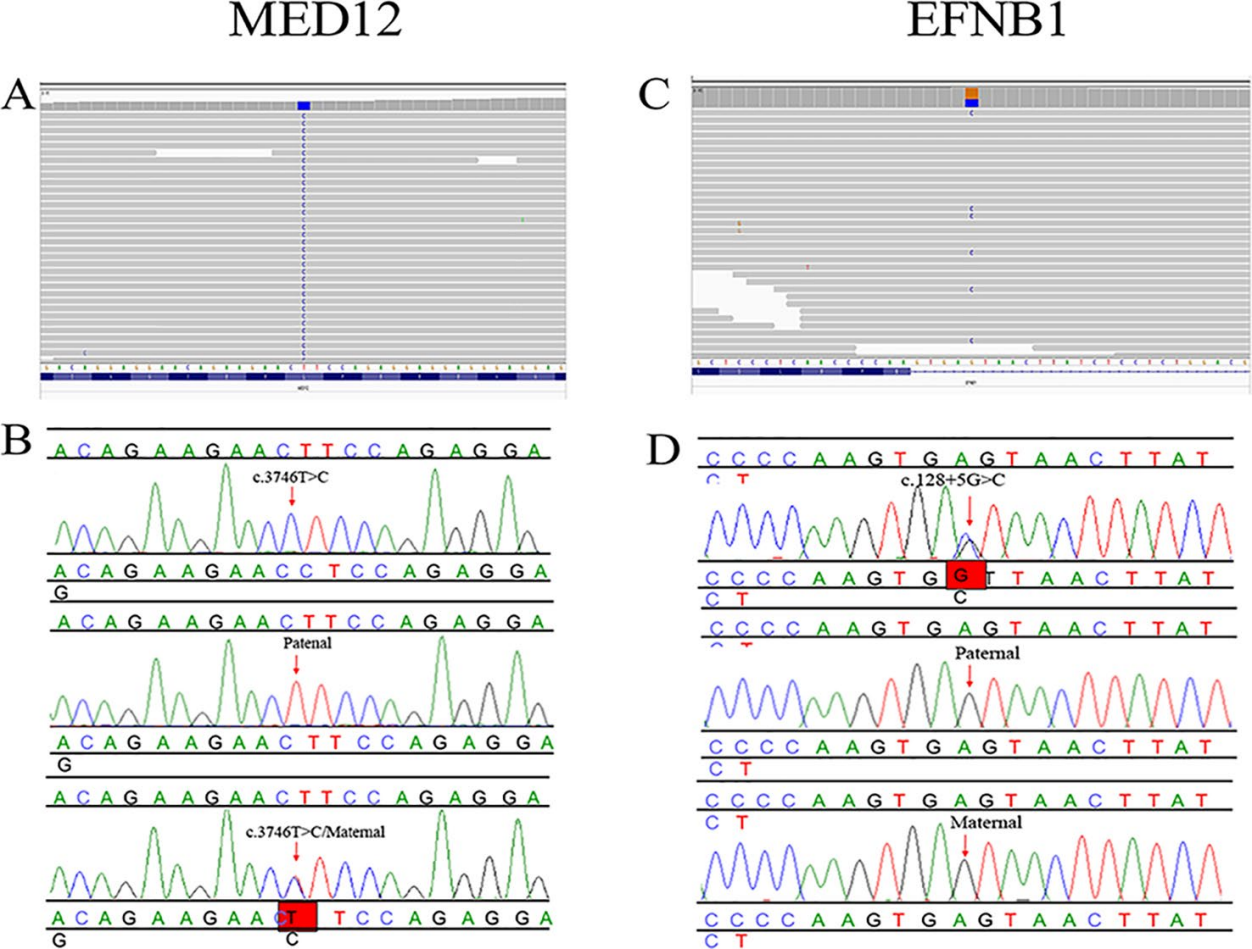
Pedigree and mutation analysis of the family. **(A**, **C)** Alignment of exon sequences to Hg19 showing SNP in exon 25 of *MED12* and intron 1 of *EFNB1*. **(B**, **D)** Chromatogram identified by Sanger sequencing. The *top* chromatograms are from the fetus, the *middle two* are from the father, and the *bottom two* are from the mother.

In proband 1, we identified 23 SNPs in callosum agenesis-related genes compared to 11 SNPs identified in proband 2. Two pathogenic mutations were identified: chrX: 70349584: T:C (c.3746T>C) in exon 25 (**Figure 2A**) and chrX: 68049752: G:C (c.128+5G>C) in intron 1 (**Figure 2C**); These two mutations were further confirmed by Sanger sequencing (**Figures 2B, D**). *MED12*: c.3746T>C of proband 1 was inherited from his healthy mother (**Figure 2B**), while *EFNB1*: c.128+5G>C of proband 2 appeared *de novo* (**Figure 2D**).

**Figure 3:**
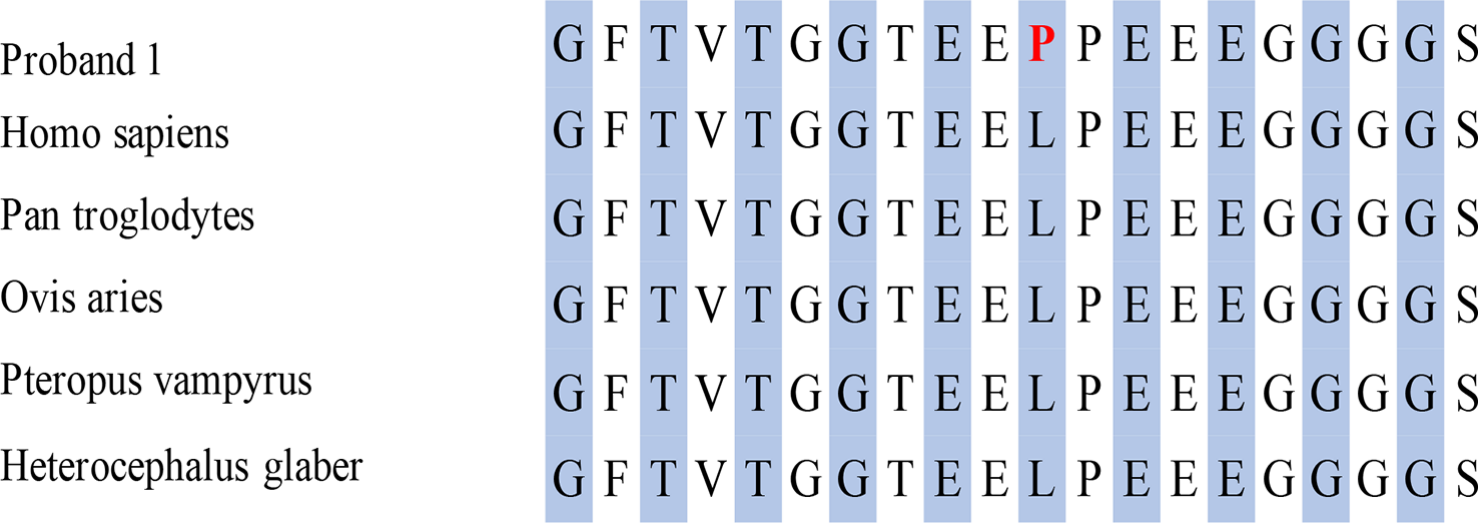
Conserved amino acid sequence of *MED12* and predicted amino acid transversion at position 3746 caused by missense mutation identified in this proband.

The T-to-C transversion at position 3746 resulted in a leucine-to-proline substitution at code1249 in the mediator complex subunit12 (*MED12*) protein (p. L1249P) (**Figure 3**), a gene which is highly conserved between different species. However, the missense mutation of *EFNB1* located in intron 1 did not result in amino acid transversion; however, it was located 5 bp behind exon 1 of *EFNB1*. The HSF database strongly supports the pathogenicity of *EFNB1*: c.128+5G>C, indicating it might influence the RNA splicing.

## Discussion

In this report, we described two families whose fetuses were diagnosed with complete agenesis of the corpus callosum. With whole-exon sequencing, we identified one hemizygous missense mutation in exon 27 of *MED12* on the X chromosome and one *de novo* heterozygous missense mutation in intron 1 of *EFNB1* on the X chromosome.

Currently, fetal MRI and ultrasound are useful tools in the diagnosis of complete ACC (cACC) in second- and third-trimester pregnancies (Tang et al., 2009). Genetic diagnosis with whole-exon sequencing offers the potential for an earlier diagnosis of cACC accompanied by the option for earlier termination of pregnancy. Though autosomal-dominant, autosomal-recessive, and X-linked patterns have been described as causes of ACC (Edwards et al., 2014), there has been no clear inheritance model found in the majority of cases, Therefore, it is likely that most cases arise from a *de novo* mutation. Some cases of partial ACC (OMIM, 304100) follow an X-linked recessive pattern caused by a mutation in the L1 cell adhesion molecule (*L1CAM*) gene on chromosome Xq28, which only affects male fetus. Additionally, Bassuk and Sherr reported a *de novo* mutation c.427T>G (S143A) of gene *PRICKLE1* in fetal agenesis of the corpus callosum without other possible mutations (Bassuk and Sherr, 2015).

In our study, we identified another X-linked recessive mutation in the *MED12* gene associated with cACC. *MED12* encodes one of the subunits of the Mediator complex, and MED12 has a role in RNA polymerase II transcription (Donnio et al., 2017). Hong et al. showed that med12-deficient zebrafish embryos showed defects in brain, neural crest, and kidney development (Hong et al., 2005). To date, at least 39 different mutations of *MED12* have been identified in patients with X-linked intellectual disability (XLID), including Lujan–Fryns syndrome (c.3020A>G, p. N1007S) [OMIM 309520], Opitz–Kaveggia or FG syndrome (FGS1)(c.2881C>T, p. R961W) [OMIM 305450], and the X-linked Ohdo syndrome [OMIM 300895] (Schwartz et al., 2007; Prontera et al., 2016).While these syndromes are caused by mutations along different positions of the *MED12* gene, they may be related with a deficient or absent corpus (Graham and Schwartz, 2013). The *EFNB1* mutation in our study follows an X-linked dominant inheritance pattern and has been mostly described in patients with craniofrontonasal syndrome (CFNS; OMIM 304110) (Wallis et al., 2008; Hogue et al., 2010). Features of the disease include frontonasal dysplasia, craniofacial asymmetry, craniosynostosis, bifid nasal tip, grooved nails, and abnormalities of the thoracic skeleton. It has been shown that *EFNB1* plays an important role in defining the position of the coronal suture (Twigg et al., 2004). Yet, to our knowledge, these two mutations have not been reported in literature. Our study expended the clinical spectrum of abnormalities associated with mutations in the X chromosome in humans. Since both of these two mutations are located in chromosome X, we inferred that male fetuses are more likely to develop complete agenesis of the corpus callosum. This finding is in accordance with a previous large cohort study on patients with agenesis of the corpus callosum that showed a slightly increased prevalence in males (56% vs. 44%) (Romaniello et al., 2017).

Since we collected samples from the expired fetuses *in utero*, there is no information regarding the intellectual function of these probands. Therefore, it was difficult to connect the mutation identified closely to intellectual disabilities, epilepsy, behavioral difficulties, or cognitive impairments in childhood or adulthood. In future studies, we are planning to carry out a large cohort analysis of agenesis of the corpus callosum with these mutations to further confirm our results and establish a scientific significance for genotype–phenotype correlation.

## Conclusion

Fast development of high-throughput sequencing provides our clinicians with a new pathway to explore the potential for genetic diagnosis of fetal ACC. Whole exon sequencing combined with Sanger sequencing demonstrated mutations of *MED12* and *EFNB1* associated with cACC and strongly implicates these two genes as one causative explanation for fetal cACC.

## Data Availability Statement

The SRA accession number for the Illumina sequencing is PRJNA549835. All other data is available on request.

## Author Contributions

YJ, Y-QQ and QL designed and wrote the paper. M-MY and Q-TZ collected clinical information, YC, F-FX and M-YD conducted the bioinformatics analysis. B-HZ revised the manuscript. MS provided the language editing. QL contributed to the study design and data interpretation and the manuscript preparation. QL is the guarantor of the work and, as such, has full access to all the data in the study and takes responsibility for the integrity of the data and the accuracy of the data analysis.

## Funding

This work was supported by the Key Subjects Group of Reproductive Medicine, School of Medicine, Zhejiang University, the National Nature Science Foundation of China (grant 81571447), and the Medical and Health Technology Program (New Technology Investigation Program) in Zhejiang Province (grant 2019321755).

## Conflict of Interest

The authors declare that the research was conducted in the absence of any commercial or financial relationships that could be construed as a potential conflict of interest.

## References

[B1] Bassuk A. G. Sherr E. H. (2015). A *de novo* mutation in PRICKLE1 in fetal agenesis of the corpus callosum and polymicrogyria. J. Neurogenet. 29, 174–177. 10.3109/01677063.2015.1088847 26727662 PMC4813514

[B2] De Ligt J. Willemsen M. H. Van Bon B. W. Kleefstra T. Yntema H. G. Kroes T. (2012). Diagnostic exome sequencing in persons with severe intellectual disability. N. Engl. J. Med. 367, 1921–1929. 10.1056/NEJMoa1206524 23033978

[B3] Donnio L. M. Bidon B. Hashimoto S. May M. Epanchintsev A. Ryan C. (2017). MED12-related XLID disorders are dose-dependent of immediate early genes (IEGs) expression. Hum. Mol. Genet. 26, 2062–2075. 10.1093/hmg/ddx099 28369444

[B4] Edwards T. J. Sherr E. H. Barkovich A. J. Richards L. J. (2014). Clinical, genetic and imaging findings identify new causes for corpus callosum development syndromes. Brain 137, 1579–1613. 10.1093/brain/awt358 24477430 PMC4032094

[B5] Fazeli A. Dickinson S. L. Hermiston M. L. Tighe R. V. Steen R. G. Small C. G. (1997). Phenotype of mice lacking functional Deleted in colorectal cancer (Dcc) gene. Nature 386, 796–804. 10.1038/386796a0 9126737

[B6] Glass H. C. Shaw G. M. Ma C. Sherr E. H. (2008). Agenesis of the corpus callosum in California 1983-2003: a population-based study. Am. J. Med. Genet. A 146A, 2495–2500. 10.1002/ajmg.a.32418 18642362 PMC2574703

[B7] Graham J. M. Jr. Schwartz C. E. (2013). MED12 related disorders. Am. J. Med. Genet. A 161A, 2734–2740. 10.1002/ajmg.a.36183 24123922 PMC3839301

[B8] Hogue J. Shankar S. Perry H. Patel R. Vargervik K. Slavotinek A. (2010). A novel EFNB1 mutation (c.712delG) in a family with craniofrontonasal syndrome and diaphragmatic hernia. Am. J. Med. Genet. A 152A, 2574–2577. 10.1002/ajmg.a.33596 20734337

[B9] Hong S. K. Haldin C. E. Lawson N. D. Weinstein B. M. Dawid I. B. Hukriede N. A. (2005). The zebrafish kohtalo/trap230 gene is required for the development of the brain, neural crest, and pronephric kidney. Proc. Natl. Acad. Sci. U.S.A. 102, 18473–18478. 10.1073/pnas.0509457102 16344459 PMC1311743

[B10] Marsh A. P. Heron D. Edwards T. J. Quartier A. Galea C. Nava C. (2017). Mutations in DCC cause isolated agenesis of the corpus callosum with incomplete penetrance. Nat. Genet. 49, 511–514. 10.1038/ng.3794 28250454 PMC5894478

[B11] Moutard M. L. Kieffer V. Feingold J. Kieffer F. Lewin F. Adamsbaum C. (2003). Agenesis of corpus callosum: prenatal diagnosis and prognosis. Childs Nerv. Syst. 19, 471–476. 10.1007/s00381-003-0781-6 12845459

[B12] Palmer E. E. Mowat D. (2014). Agenesis of the corpus callosum: a clinical approach to diagnosis. Am. J. Med. Genet. C Semin. Med. Genet. 166C, 184–197. 10.1002/ajmg.c.31405 24866859

[B13] Prontera P. Ottaviani V. Rogaia D. Isidori I. Mencarelli A. Malerba N. (2016). A novel MED12 mutation: Evidence for a fourth phenotype. Am. J. Med. Genet. A 170, 2377–2382. 10.1002/ajmg.a.37805 27312080

[B14] Romaniello R. Marelli S. Giorda R. Bedeschi M. F. Bonaglia M. C. Arrigoni F. (2017). Clinical characterization, genetics, and long-term follow-up of a large cohort of patients with Agenesis of the Corpus Callosum. J. Child Neurol. 32, 60–71. 10.1177/0883073816664668 27683483

[B15] Santo S. D’antonio F. Homfray T. Rich P. Pilu G. Bhide A. (2012). Counseling in fetal medicine: agenesis of the corpus callosum. Ultrasound Obstet. Gynecol. 40, 513–521. 10.1002/uog.12315 23024003

[B16] Schwartz C. E. Tarpey P. S. Lubs H. A. Verloes A. May M. M. Risheg H. (2007). The original Lujan syndrome family has a novel missense mutation (p.N1007S) in the MED12 gene. J. Med. Genet. 44, 472–477. 10.1136/jmg.2006.048637 17369503 PMC2597996

[B17] Smith K. M. Ohkubo Y. Maragnoli M. E. Rasin M. R. Schwartz M. L. Sestan N. (2006). Midline radial glia translocation and corpus callosum formation require FGF signaling. Nat. Neurosci. 9, 787–797. 10.1038/nn1705 16715082

[B18] Tang P. H. Bartha A. I. Norton M. E. Barkovich A. J. Sherr E. H. Glenn O. A. (2009). Agenesis of the corpus callosum: an MR imaging analysis of associated abnormalities in the fetus. AJNR Am. J. Neuroradiol. 30, 257–263. 10.3174/ajnr.A1331 18988682 PMC7051410

[B19] Twigg S. R. Kan R. Babbs C. Bochukova E. G. Robertson S. P. Wall S. A. (2004). Mutations of ephrin-B1 (EFNB1), a marker of tissue boundary formation, cause craniofrontonasal syndrome. Proc. Natl. Acad. Sci. U.S.A. 101, 8652–8657. 10.1073/pnas.0402819101 15166289 PMC423250

[B20] Wallis D. Lacbawan F. Jain M. Der Kaloustian V. M. Steiner C. E. Moeschler J. B. (2008). Additional EFNB1 mutations in craniofrontonasal syndrome. Am. J. Med. Genet. A 146A, 2008–2012. 10.1002/ajmg.a.32388 18627045 PMC2774847

